# Helminth Parasites of *Rhombomys opimus* from Golestan Province, Northeast Iran

**Published:** 2013

**Authors:** B Kamranrashani, EB Kia, I Mobedi, M Mohebali, Z Zarei, Gh Mowlavi, H Hajjaran, MR Abai, M Sharifdini, Z Kakooei, H Mirjalali, S Charedar

**Affiliations:** 1 Department of Medical Parasitology and Mycology, School of Public Health, Tehran University of Medical Sciences, Tehran, Iran; 2 Center for Research of Endemic Parasites of Iran (CREPI), Tehran University of Medical Sciences, Tehran, Iran; 3 Department of Medical Entomology and Vector Control, School of Public Health, Tehran University of Medical Sciences, Tehran, Iran

**Keywords:** *Rhombomis opimus*, Helminths, Parasites, Iran

## Abstract

**Background:**

The aim of the study was to determine the helminthic species occurring in great gerbil *Rhombomys opimus* collected from Maraveh Tappeh, Golestan Province, northeast Iran.

**Methods:**

During 2010-2011, a total of 77 *R. opimus* were captured from rural areas of Maraveh Tappeh, Golestan Province, using Sherman live traps and examined for infectivity with any larva or adult stages of helminthic parasites.

**Results:**

Overall, 63 *R. opimus* (81.8%) were found infected with different helminthic species. The rate of infectivity with each species was as follows: *Trichuris rhombomidis* 31.2%, *Trichuris muris* 32.5%, *Trichuris* spp. 10.4%, *Syphacia muris* 2.6%, *Dipetalonema viteae (Acanthocheilonema viteae*) 37.7%, *Skrjabinotaenia lobata* 15.6%, *Hymenolepis (=Rodentolepis) nana fraterna* 5.2%, and *Taenia endothoracicus* larva 1.3%.

**Conclusion:**

*R. opimus* is host for several species of cestodes and nematodes in the study area. The high rate of infectivity with *D. viteae* indicates the susceptibility of these gerbils to this filarial nematode. Synchronous infections occurred up to four species of helminthes in one host.

## Introduction

The great gerbil *Rhombomis opimus* (Lichtenstein 1832) (Rodentia: gerbillinae) has a vast distribution range from Iran through Pakistan, Afghanistan via Kazakhstan to China and Mongolia (1). They are most abundant in sand and clay deserts (2), and in some areas they are widely regarded as a pest species (1). In Iran, they are distributed throughout south, central and northeast of the country, including Golestan Province (3) in north of the country. Since long time ago *R. opimus* is known as a principal natural reservoir of zoonotic cutaneous leishmaniasis (ZCL) in Iran. One of these natural foci is located in Golestan Province (4). In spite of distribution of *R. opimus* in this province, and adjacent of their colonies with rural housings, no attention has been paid on the other parasitic agents in this rodent. Actually, there are only rare reports on occurrence of helminthic parasites in this rodent in Iran; these reports are from central parts of the country, including Isfahan (5) and on a few numbers from Kashan (6).

Since this gerbil is capable to harbor a wide variety of helminthic parasites especially cestodes and nematodes species (1), as definitive or intermediate host, this study was aimed to determine the helminthic species occurring in *R. opimus* in rural area of this province, where this gerbil is in close association with human settlements.

## Material & Methods

### Study site

**Fig. 1:**
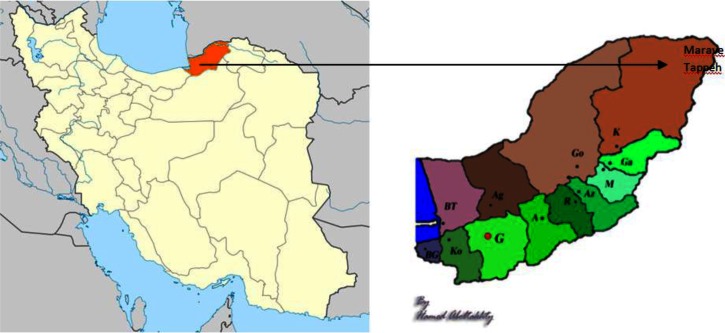
Map of the study area; Left: Map of Iran, Right: Map of Golestan Province

Golestan Province (36° 83’ 93" N and 54° 44’ 44" E) is located in the north east of Iran, south of the Caspian Sea, with Gorgan as capital. Golestan has an area of 20,380 km^2^. Three different climates exist in the region, including plain moderate, mountainous, and semi-arid. The study site, Maraveh Tappeh (Fig. 1), is a county in this province. It is located between two mountain ranges, having steppe vegetation. It is in adjacent to Turkmenistan Country from north and North Khorasan Province from east. The climate of the county is semi-arid (7).

### Sampling

Samples of *R. opimus* were part of a collection for a molecular research on *Leishmania* species in this area. Samplings were carried out during 2010-2011, using Sherman live traps, baited with walnut, cucumber or tomato. Traps were placed near the burrows of *R. opimus* tunnels from early sunrise till evening, with an inspection around nine o'clock for collection of captured rodents and resetting the traps. Rodents were transported to the field laboratory in Maraveh Tappeh, keeping in cool and shaded place with sufficient food, prior to shipping to the Animal Unit of the School of Public Health, Tehran University of Medical Sciences, where animals where anesthetized and bled. This study was approved by Ethics Committee of Tehran University of Medical Sciences.

### Rodents’ examination and Parasites identification

Thin and thick blood smears were prepared, for further Giemsa staining and microscopical observation. Soon after death, the carcass of each *R. opimus* was carefully dissected and every organ was examined separately under stereomicroscope for the presence of any helminthic larva or adult. Subcutaneous tissue was observed macroscopically for the presence of adult filaria. Recovered parasites were removed and relaxed in warm saline prior to gradual preservation in 70% ethanol. Samples of cestode parasites and *Trichuris* were tied between two proper glasses before immersing in the preservative. Before microscopical examination, parasites were either cleared in lactophenol or stained by carmine alum, dehydrating in graded series of ethanol alcohol, clearing in xylene and mounting in canada balsam. For each animal, formalin ether sedimentation technique was also performed on feces and examined for the presence of helminth egg or larva.

Morphological and morphometrical characteristics of helminthes were characterized with the aid of a calibrated microscope, equipped with camera lucida drawing tube. For species identification valid systematic keys including different volumes of K I Skrjabin et al., as well as Yamaguti S (8) were used. Data processing and statistical analysis were performed using SPSS version 11.5. A *P*-value of <0.05 was considered as significant difference.

## Result

Overall, 77 *R. opimus* were examined for infectivity with helminth parasites. Table 1 represents the rate of infectivity with different species of helminthes according to the sex of *R. opimus* and the organ involved. If different species of *Trichuris* are pooled and considered altogether, the genus *Trichuris* would be the most prevalent one (62.3%).

**Table 1:** Frequency and Prevalence of helminth parasites in *Rhombomys opimus* from Golestan Province according to the sex of the rodent and different organs

Organ	Helminth species	Rodent sex			
		Male (N = 65)		Female (N = 12)		Total (N = 77	
		No.	%	No.	%	No.	%
Omentum	*Taenia endothoracicus larva*	1	1.5	-	-	1	1.3
Small intestine	*Hymenolepis nana fraterna*	1	1.5	3	25	4	5.2
	*Skrjabinotaenia lobata*	10	15.4	2	16.7	12	15.6
Large intestine and cecum	*Trichuris rhombomidis*	21	32.3	3	25	24	31.2
	*Trichuris muris*	25	38.5	-	-	25	32.5
	*Trichuris* sp.	2	3.1	6	50	8	10.4
	*Syphacia muris*	-	-	2	16.7	2	2.6
Subcutaneous and blood	Dipetalonema viteae	26	40	3	25	29	37.7
Total Infection*		53	81.5	10	83.3	63	81.8

* There were some cases of co-infection with two or more parasites.

**Fig. 2:**
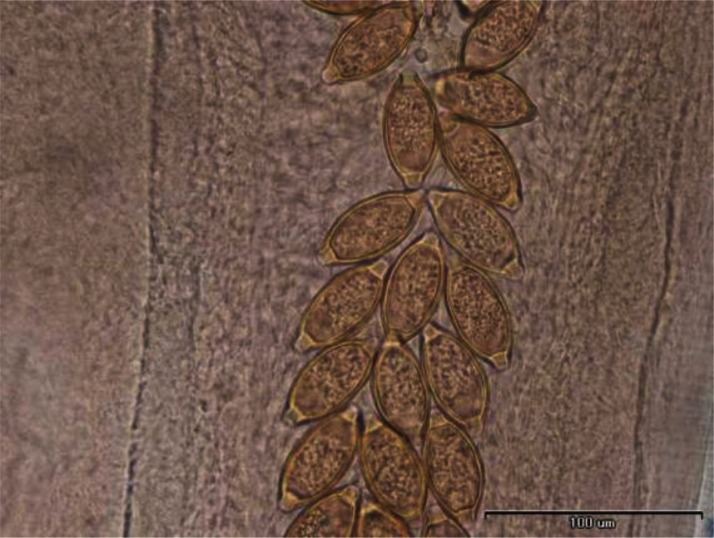
Eggs of *Trichuris* sp

**Fig. 3:**
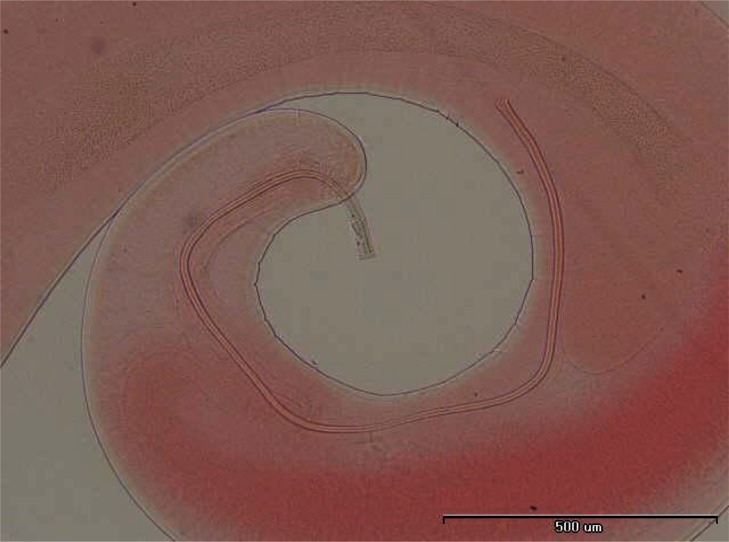
*Trichuris rhombomidis* posterior end of male

**Fig. 4:**
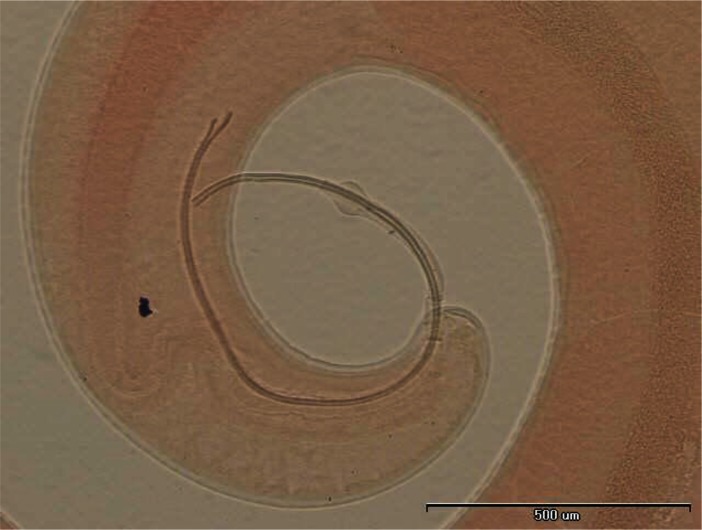
*Trichuris muris* posterior end of male

In some individuals, especially females of *R. opimus* only female nematodes were present and based on the morphological criteria species determination was not accurate; therefore, in such cases the species were registered as *Trichuris* sp. (Fig. 2). However, among those gerbils harboring male *Trichuris*, species identification was performed and accordingly 31.2% and 32.5% of the gerbils were found infected with *Trichuris rhombomidis* (Fig. 3) and *Trichuris muris* (Fig. 4), respectively. In 11.7% also co-infectivity with both species was found (not shown in the table).

**Fig. 5:**
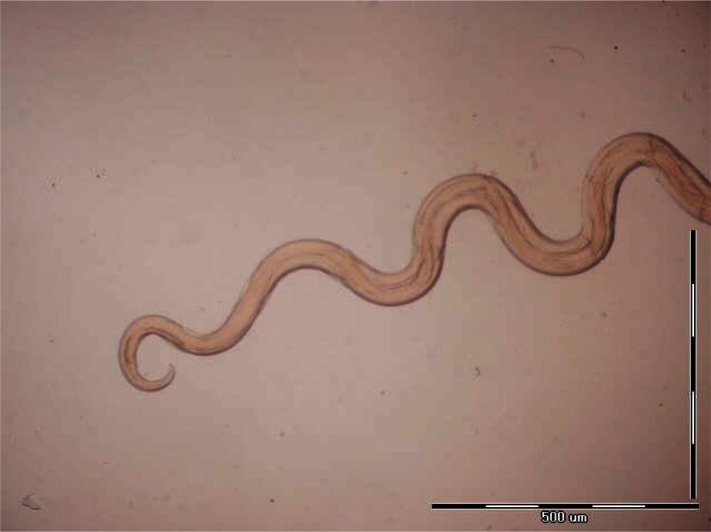
Posterior end of *Dipetalonema viteae* male

**Fig. 6:**
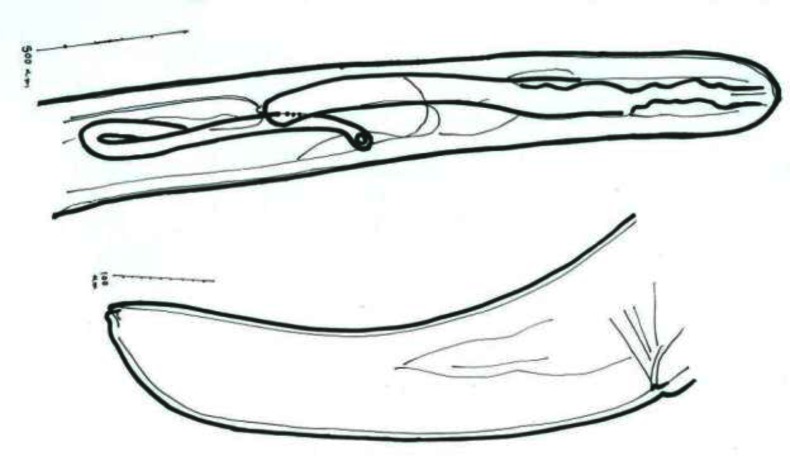
Camera lucida drawings of anterior and posterior ends of *Dipetalonema viteae* female

**Fig. 7:**
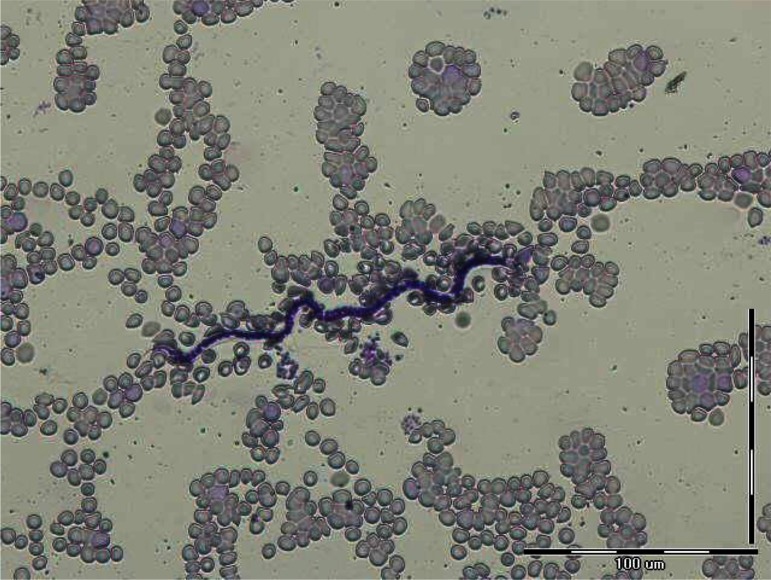
*Dipetalonema viteae* microfilariae in peripheral blood of *Rhombomis opimus*

**Fig. 8:**
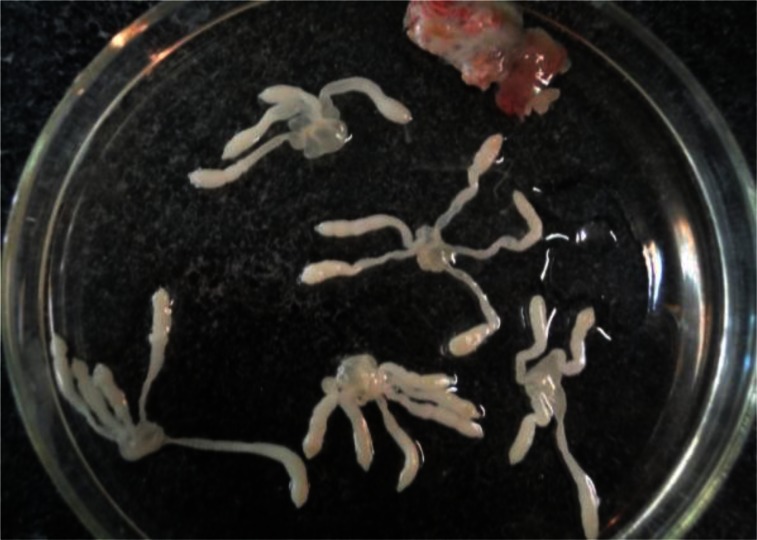
Larvae of *Taenia endothoracicus*

**Fig. 9:**
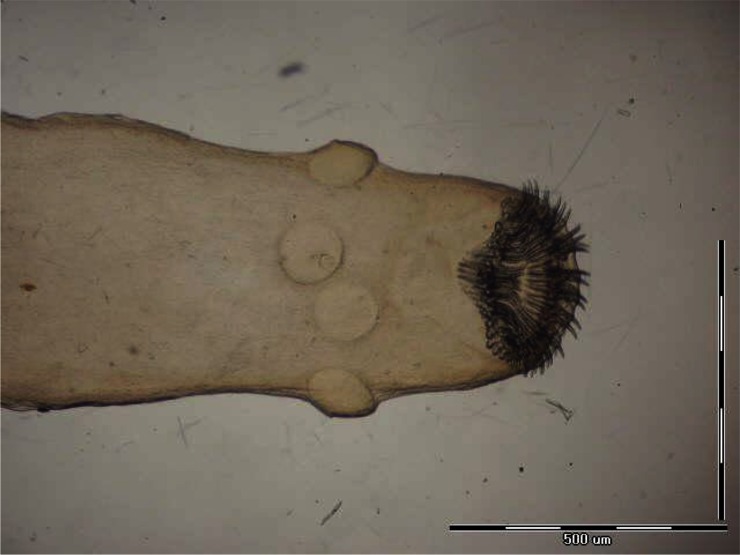
Scolex of *Taenia endothoracicus* larva

**Fig. 10:**
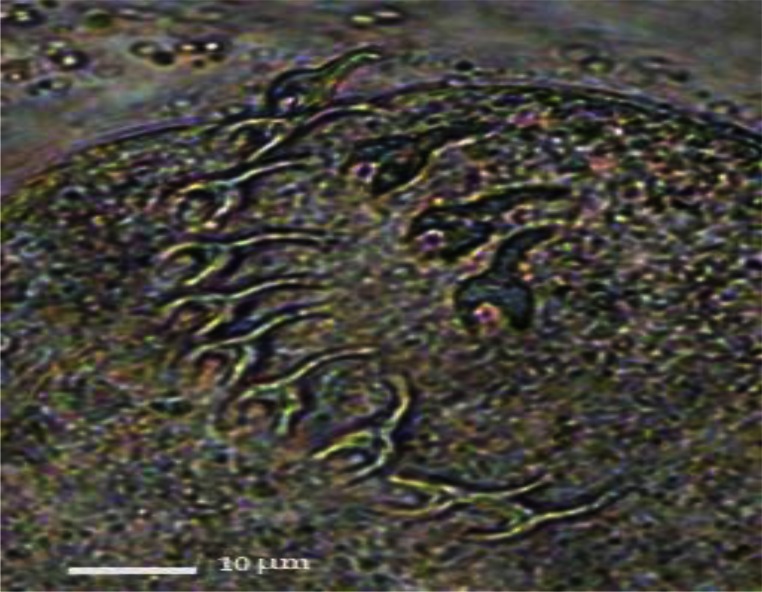
Hooks of *Hymenolepis nana fraterna*

**Fig. 11:**
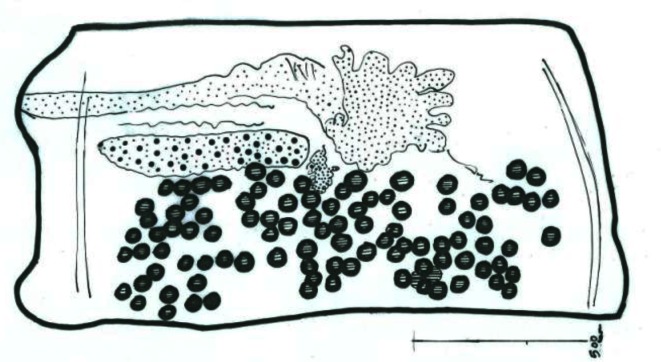
Camera lucida drawing of *Skrjabinotaenia lobata* mature proglotid

As Table 1 shows, the most prevalent helminth species was *Dipetalonema* (*Acanthocheilonema*) *viteae* (37.7%). In infected *R. opimus*, the adult *D. viteae* (Figs. 5 and 6) were found subcutaneously and microfilaria in the peripheral blood (Fig. 7). Respect to the cestodes occurring in *R. opimus*, three species were identified including *Taenia endothoracicus* larva (Figs. 8 and 9) in omentum (1.3%) and *Hymenolepis* (*= Rodentolepis*) *nana fraterna* (5.2%) (Fig. 10) and *Skrjabinotaenia lobata* (15.6%) (Fig. 11) in small intestine. Statistically, *H. nana fraterna* and *T. muris* were significantly more prevalent in female (*P*= 0.01) and male (*P*= 0.006), respectively. For, other species no sex ratio difference was found.

Synchronous infections occurred up to four species of helminthes in one rodent. Percentages of infectivity with one, two, three and four species of helminthes in infected gerbils were 50.6%, 20.8%, 9.1%, and 1.3%, respectively. No sex-related statistical difference was there respect to the variety of helminth species.

## Discussion

*Rhombomys opimus* inhabits the desert of central Asia, from the Caspian Sea to southern Mongolia and north-central China (2). In Iran, it is known as the most important reservoir for ZCL (4). One of the provinces in Iran in which *R. opimus* is abundant is Golestan Province (3). In this study the helminth parasites of 77 *R. opimus* collected from rural areas of this province were identified. In general 81.8% of the gerbils were found infected with at least one species of helminthes. Genus *Trichuris* was found in 62.3% of *R. opimus*. Between the two identified *Trichuris* species, *T. muris* (32.5%) has been frequently reported from different species of rodents in the country (6, 9, 10); but *T*. *rhombomidis* (31.2%) has been rarely reported (11). In a study on helminth fauna of *R. opimus* in Uzbekistan, *T. rhombomidis* comprised the most frequent heminth parasite (in 78.4%) in Tashkent and Samarkand districts in this country (12).

One of the other prevalent helminth species in the current study was *D. viteae* with the rate of 37.7% infectivity. Although the males were more infected than females (40% vs. 25%), but statistically, the difference was not significant. This is probably due to low sample size of trapped females. There is only one previous documented record on occurrence of this parasite in Iran, back to 1967 from *Meriones persicus* in Tehran and Ghazvin (13). Those authors declared that soft tick *Ornithodorus* was the vector of this nematode. The occurrence of *D. viteae* from *R. opimus* in this study constitutes new host record in the country.

Among cestodes occurring in *R. opimus*, *H. nana fraterna* has been frequently reported from different species of rodents in the country (5, 6, 10, 11, 14–16), as well as laboratory animals (17), however, the reports on occurrence of the two other mentioned cestodes are rare in Iran. *T*. *endothoracicus* larva has been reported from *Meriones* species including *Meriones lybicus* (5, 18), and *Meriones persicus* (14); and *S. lobata* from *M. persicus* (15) , and *Tatera indica* (11). Therefore, occurrences of *T*. *endothoracicus* larva and *S. lobata* in *R. opimus* in this study constitute records in new host species in Iran.

Considering sex-related distribution, statistical analysis revealed significant differences between males and females of *R. opimus* and infectivity with *H. nana fraterna* and *T. muris*. For *H. nana* females were more infected than males (*P*=0.01). Similar result has also been observed in brown rats *Rattus norvegicus* infected with this cestode (19). This is most probably due to direct life cycle of this parasite and feasible transmission of eggs from adult females to their newborns during lactating and nursing. On the contrary, *T. muris* was significantly higher in males of *R. opimus* (*P*=0.006). This finding is coincident with the report on significantly higher prevalence of *T. muris* in males than females in *R. norvegicus* from urban habitats of Belgrade area (Serbia) (20). According to the results of this study, *R. opimus* is host for variety of cestodes (three species) and nematodes (at least five species) in the study area. Synchronous infections were found up to four species of helminthes in single host. This is coincidence with the result of Kataranovski et al. (20) who reported no more than four parasite species in *R. norvegicus*.

## Conclusion

A high rate of infectivity with helminth parasites (81.8%) was found in *R. opimus* in the study area. The most prevalent parasites were genus *Trichuris* and *D. viteae*. The occurrence of *D. viteae*, *T. endothoracicus* larva and *S. lobata* in *R. opimus* constituted new host records in Iran. The high rate of infectivity with *D. viteae* indicates the susceptibility of these gerbils to this filarial nematode. Further studies on *D. viteae* are needed to determine its natural vector in the area, as well as the role of congenital transmission of this parasite to *R. opimus*.
